# Fractional Derivative Modification of Drude Model

**DOI:** 10.3390/s21154974

**Published:** 2021-07-22

**Authors:** Karol Karpiński, Sylwia Zielińska-Raczyńska, David Ziemkiewicz

**Affiliations:** Institute of Mathematics and Physics, UTP University of Science and Technology, 85-796 Bydgoszcz, Poland; karol.karpinski@utp.edu.pl (K.K.); sziel@utp.edu.pl (S.Z.-R.)

**Keywords:** digital filters, electrodynamics, electromagnetic propagation, finite difference methods, optical surface waves, physics computing, propagation

## Abstract

A novel, two-parameter modification of a Drude model, based on fractional time derivatives, is presented. The dielectric susceptibility is calculated analytically and simulated numerically, showing good agreement between theoretical description and numerical results. The absorption coefficient and wave vector are shown to follow a power law in the frequency domain, which is a common phenomenon in electromagnetic and acoustic wave propagation in complex media such as biological tissues. The main novelty of the proposal is the introduction of two separate parameters that provide a more flexible model than most other approaches found in the literature. Moreover, an efficient numerical implementation of the model is presented and its accuracy and stability are examined. Finally, the model is applied to an exemplary soft tissue, confirming its flexibility and usefulness in the context of medical biosensors.

## 1. Introduction

Fractional calculus, the branch of mathematics devoted to studying non-integer-order derivatives and integrals, has become an increasingly popular tool for analysis of various problems in physics, ranging from quantum mechanics and cosmology [1] to electric circuits [2] and electromagnetic wave propagation [3]. An extensive review of recent developments is presented in [4].

The application of fractional derivatives in theoretical [5] and numerical [6] descriptions of mechanical waves has been extensively studied, including the propagation of acoustic waves [7]. One of the key motives behind the development of fractional models is the observation that wave attenuation in many systems follows a power law with a non-integer exponent [8,9], which cannot be described with the use of standard time domain partial differential equations. Such power laws are especially prevalent in acoustic wave propagation in biological tissues [10]. Furthermore, many physical media exhibit hereditary features [11], where some physical property is dependent on the history of its previous values. The fractional derivative provides a tool with which to analyze such systems that carry information about their present as well as past states [12,13,14].

Motivated by these developments, we propose an extension of the Drude model, which is one of the basic tools for describing the electric permittivity of metals. To better fit the experimental observations in complex media, several extensions of the Drude model have been proposed, ranging from the introduction of frequency-dependent parameters [15] to the use of fractional derivatives [16]. These models can be used in various numerical approaches to calculate the wave propagation in such media. One of the most popular methods is the finite-difference time-domain (FDTD) [17]. Usually, a direct numerical implementation of fractional derivatives is problematic. For the so-called Cole–Cole model, which is a popular choice for the description of soft tissue, multiple simplifications have been proposed [18,19]. Another similar relation is the Havriliak–Negami medium, which is also applied to soft tissues as well as to liquid dielectrics and polymers [20]. Fractional-derivative-based models are particularly valuable in the description of electromagnetic wave biosensors. Depending on the applications, these devices are designed in a wide range of frequencies, from radio frequencies up to the optical spectrum [21] and use the permittivity spectrum of the sample as a unique dielectric signature that can be utilized for sensing purposes. For proper description of the multiple polarization mechanisms that contribute to the total permittivity of the tissue, one has to apply a complex model with many relaxation terms [21]. Fractional derivatives offers a simpler approach to modeling the electromagnetic response of such media. Finally, it should be noted that the results are also applicable to acoustic wave propagation in soft tissues and the related medical imaging sensors [22,23,24].

The approach presented here introduces two fractional derivatives to the equations of motion describing the polarization of the medium, resulting in a more general and flexible model. It is shown that by using the truncated Grunwald–Letnikov derivative [25], one can achieve a good accuracy while retaining low calculation complexity, roughly on the same level as in [18,19,20]. The detailed, novel implementation of the medium description in FDTD is presented and the accuracy of simulation results is discussed. In addition to the comparison between analytical and FDTD results, an example fit to the experimental data regarding soft tissue [26] is presented, demonstrating that our approach offers more flexibility than a regular Drude model.

## 2. Drude Model

We assume that the medium (usually a metal) contains some concentration *n* of free charges *e*, of effective mass *m*. The equation describing their motion under the influence of external field *E* is

(1) $$m\ddot{\vec{r}}+\Gamma \dot{\vec{r}}=e\vec{E},$$

where $\vec{r}$ is the charge position and Γ describes dissipative processes. By introducing the polarization vector $\vec{P}=ne\vec{r}$, the damping constant γ=Γ/m and the plasma frequency $\omega_{p}^{2}=ne^{2}/m$, one obtains

(2) $$\ddot{P}+\gamma \dot{P}=\omega_{p}^{2}E$$

which, in the time domain, for a harmonic wave $E=E_{0}e^{-i\omega t}$, $P=P_{0}e^{-i\omega t}$ yields

(3) $$P_{0}=\chi \left(\omega \right)E_{0}=\frac{-\omega_{p}^{2}}{\omega^{2}+i\gamma \omega }E_{0},$$

which is a standard expression for modeling the electric susceptibility of metals [15]. In our modified model, we introduce the fractional derivative operator D to the Equation (2) in all instances where the time derivative is used. In particular, we transform the Equation (2) into the form

(4) $$\gamma_{\alpha }D^{\alpha +1}P+\gamma_{\beta }D^{\beta }P=\omega_{p}^{2}E,$$

where $D^{\beta }$ denotes fractional derivatives of the order β and α, and β are real parameters. In particular, we use a Grunwald–Letnikov derivative [25]. For a polynominal function of time $f\left(t\right)=t^{n}$, $D^{\alpha }f=0$ for α>n; for harmonic waves considered here, $D^{\alpha }e^{i\omega t}={\left(i\omega \right)}^{\alpha }e^{i\omega t}$. Standard relation (2) is obtained from (4) by taking $\gamma_{\alpha }=\alpha =\beta =1$. In the frequency domain, the above relation leads to the susceptibility

(5) $$\chi \left(\omega \right)=\frac{P_{0}}{E_{0}}=\frac{\omega_{p}^{2}}{\gamma_{\alpha }{\left(-i\omega \right)}^{\alpha +1}+\gamma_{\beta }{\left(-i\omega \right)}^{\beta }}.$$

Note that the constants $\gamma_{\alpha }$, $\gamma_{\beta }$ ensure the proper dimensionality of the equation, e.g., $\left[\gamma_{\alpha }\omega^{\alpha +1}\right]=\left[\omega^{2}\right]$ and $\left[\gamma_{\beta }\omega^{\beta }\right]=\left[\omega^{2}\right]$, so that χ remains a dimensionless quantity. Note that we assume that the frequency ω>0, so that electrostatic fields are not modeled. Thus, the polarization *P* that is induced by a changing electric field is also always changing in time.

In contrast to other extensions of the Drude model, where the dissipation constant and/or plasma frequency are frequency-dependent quantities [15], we introduce additional frequency dependence by directly modifying the exponent of the frequency. The susceptibility is connected with the complex medium permittivity

(6) $$\epsilon \left(\omega \right)=\epsilon_{\infty }+\chi \left(\omega \right)+\frac{i\sigma }{\omega \epsilon_{0}},$$

where σ is the conductivity, $\epsilon_{0}$ is the vacuum permittivity and $\epsilon_{\infty }$ is the permittivity in the limit of a very high frequency. Real and imaginary parts of ϵ are responsible for the dispersion and absorption, respectively.

The Equation (4) can be easily adapted to model the Debye relation [18]

(7) $$\epsilon \left(\omega \right)=\epsilon_{\infty }+\frac{\delta \epsilon }{1+i\omega \tau },$$

by taking β=0, α=0, $\omega_{p}^{2}<0$, $\gamma_{b}=1$. Usually, multiple terms as in Equation (7) are used to model various relaxation processes [21]. However, due to the use of two separate parameters α, β, the Equation (5) provides more flexibility than a simple Debye model. In fact, it can be used to approximate some other fractional-derivative-based models. In particular, the Cole–Cole model is given by [18]

(8) $$\epsilon \left(\omega \right)=\epsilon_{\infty }+\frac{\Delta \epsilon }{1+{\left(i\omega \tau \right)}^{1-\alpha }}+\frac{\sigma }{i\omega \epsilon_{0}}.$$

One can have a direct correspondence between Equations (5) and (8) by taking β=0. However, in the Havriliak–Negami model [20]

(9) $$\epsilon \left(\omega \right)=\epsilon_{\infty }+\frac{\Delta \epsilon }{{\left(1+{\left(i\omega \tau \right)}^{\alpha }\right)}^{\beta }},$$

there are two parameters that do not correspond exactly to α, β in Equation (4).

## 3. Numerical Implementation

The finite-difference time-domain method (FDTD) consists of dividing the simulation space into grid points and calculating the values of the electric and magnetic fields at those points with evolution equations derived directly from Maxwell’s equations, with some finite time step Δt [17]. Due to the facts that the algorithm is based on first principles, has well-known sources of numerical errors, and is easy to implement in parallel computing, it is one of the leading tools for analysis of complex optical and plasmonic systems. One of the methods used to include complex media in FDTD simulations is the auxiliary differential equation (ADE) approach [27]; the medium polarization *P* is computed by numerically solving a partial differential equation describing its evolution in time. For the regular Drude model, one can derive the evolution equation for *P* from Equation (2). In the FDTD scheme, one has a set of discrete values of polarization $P_{t}$, t=1,2,3⋯. To obtain the first-order approximation of the first time derivative, one can use the relations

(10) $$\begin{array}{c} {\dot{P}}_{t+1/2}\approx \frac{P_{t+1}-P_{t}}{\Delta t},\\ {\dot{P}}_{t-1/2}\approx \frac{P_{t}-P_{t-1}}{\Delta t},\\ \dot{P_{t}}\approx \frac{1}{2}\left({\dot{P}}_{t+1/2}+{\dot{P}}_{t-1/2}\right). \end{array}$$

In a similar manner, one can define the second derivative as

(11) $$\ddot{P_{t}}\approx \frac{1}{\Delta t}\left({\dot{P}}_{t+1/2}-{\dot{P}}_{t-1/2}\right).$$

In our modified approach, the first step is to define the fractional derivative operator; from the standpoint of computation with a discrete time step Δt, the most convenient definition is the truncated Grunwald–Letnikov derivative [25]

(12) $$D^{\alpha }f\left(t\right)=\lim_{\Delta t\to 0}\frac{1}{\Delta t^{\alpha }}\sum_{k=0}^{N}{\left(-1\right)}^{k}\frac{\Gamma (\alpha +1)f(t-k\Delta t)}{\Gamma (k+1)\Gamma (\alpha -k+1)},$$

where in numerical implementation Δt is set to some finite value and *N* is a suitably large number such that the components of the sum are negligibly small. Note that the left-sided derivative is used, where only previous values of f(t) are needed. In other words, the model is causal. In terms of discrete values of *P*, one obtains

(13) $$\begin{array}{ccc} & & D^{\alpha }P_{t-\frac{1}{2}}=\frac{1}{\Delta t^{\alpha }}\sum_{k=0}^{N}{\left(-1\right)}^{k}\frac{\Gamma (\alpha +1)}{\Gamma (k+1)\Gamma (\alpha -k+1)}P_{t-k},\\ & & =\frac{1}{\Delta t^{\alpha }}\sum_{k=0}^{N}\alpha_{k}P_{t-k}. \end{array}$$

By applying the above definition and the averaging procedure in Equations (10) and (11) to the Equation (4), one obtains the relations for derivatives

(14) $$\begin{array}{ccc} & & D^{\alpha +1}P_{t}=\frac{1}{\Delta t\Delta t^{\alpha }}\sum_{k=0}^{N}\alpha_{k}P_{t-k+1}-\alpha_{k}P_{t-k}\\ & & D^{\beta }P_{t}=\frac{1}{2\Delta t^{\beta }}\sum_{k=0}^{N}\beta_{k}P_{t-k+1}+\beta_{k}P_{t-k} \end{array}$$

and the equation of motion

(15) $$\begin{array}{ccc} & & \frac{\gamma_{\alpha }}{\Delta t\Delta t^{\alpha }}\sum_{k=0}^{N}\alpha_{k}\left(P_{t-k+1}-P_{t-k}\right)\\ & & +\frac{\gamma_{\beta }}{2\Delta t^{\beta }}\sum_{k=0}^{N}\beta_{k}\left(P_{t-k}+P_{t-k+1}\right)-\omega_{p}^{2}E_{t}=0.\; \end{array}$$

After expanding and rearranging the terms, one obtains the evolution relation

(16) $$\begin{array}{ccc} P_{t+1}=-\frac{\frac{\gamma_{\alpha }\left(\alpha_{1}-\alpha_{0}\right)}{\Delta t\Delta t^{\alpha }}+\frac{\gamma_{\beta }\left(\beta_{0}+\beta_{1}\right)}{2\Delta t^{\beta }}}{\frac{\gamma_{\alpha }\alpha_{0}}{\Delta t\Delta t^{\alpha }}+\frac{\gamma_{\beta }\beta_{0}}{2\Delta t^{\beta }}} & P_{t} & \\ -\frac{\frac{-\gamma_{\alpha }\alpha_{1}}{\Delta t\Delta t^{\alpha }}+\frac{\gamma_{\beta }\beta_{1}}{2\Delta t^{\beta }}}{\frac{\gamma_{\alpha }\alpha_{0}}{\Delta t\Delta t^{\alpha }}+\frac{\gamma_{\beta }\beta_{0}}{2\Delta t^{\beta }}} & P_{t-1} & \\ -\frac{\frac{\gamma_{\alpha }}{\Delta t\Delta t^{\alpha }}}{\frac{\gamma_{\alpha }\alpha_{0}}{\Delta t\Delta t^{\alpha }}+\frac{\gamma_{\beta }\beta_{0}}{2\Delta t^{\beta }}}\sum_{k=2}^{N}\alpha_{k} & \left(P_{t-k+1}-P_{t-k}\right) & \\ -\frac{\frac{\gamma_{\beta }}{2\Delta t^{\beta }}}{\frac{\gamma_{\alpha }\alpha_{0}}{\Delta t\Delta t^{\alpha }}+\frac{\gamma_{\beta }\beta_{0}}{2\Delta t^{\beta }}}\sum_{k=2}^{N}\beta_{k} & \left(P_{t-k+1}+P_{t-k}\right) & \\ +\frac{\omega_{p}^{2}}{\frac{\gamma_{\alpha }\alpha_{0}}{\Delta t\Delta t^{\alpha }}+\frac{\gamma_{\beta }\beta_{0}}{2\Delta t^{\beta }}} & E_{t} & . \end{array}$$

which relates the new value of $P_{t+1}$ to the current value of $P_{t}$, the electric field $E_{t}$ and the history $P_{t-k}$. For the case of α=β=1, $\alpha_{k}=\beta_{k}=0$ for k>1 the relation simplifies to the standard form presented in [27].

## 4. Results

### 4.1. Calculation of Susceptibility

**Figure 1:**
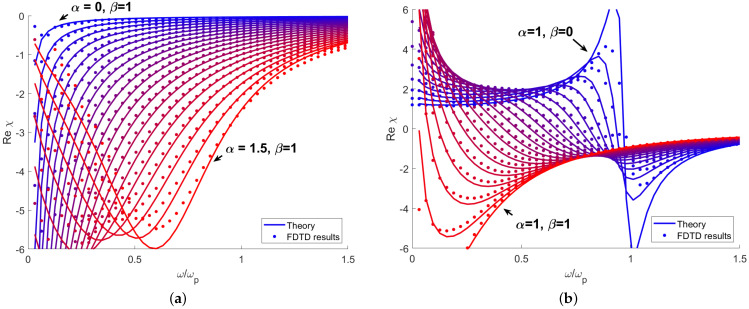
Real part of susceptibility calculated analytically from (5) and numerically for various values of (**a**) α and (**b**) β.

Figure 1 shows the real part of the susceptibility calculated from the analytical solution (5) and obtained from FDTD simulation. The medium parameters are set to $\omega_{p}=0.3$, $\gamma_{\beta }=0.1$, $\gamma_{\alpha }=1$. In Figure 1a the value of β is set to 1 and 0≤α≤1.5. Like in the standard Drude model, the susceptibility is negative and for α≤1, χ→−∞ when ω→0. For higher values of α, a distinct minimum of susceptibility occurs. One can see that the accuracy is excellent in the medium frequency range, e.g., $\omega \approx \omega_{p}$; at the low frequency limit, the large susceptibility corresponds to short wavelengths, which approaches the finite spatial step of the simulation. Additionally, waves are highly absorbed in this region, further decreasing prediction accuracy. In the very high frequency regime, accuracy is limited by the fact that the wave period approaches the finite time step. In this region, χ→0 and the value of α has a significant effect on the speed at which this asymptote is approached. Figure 1b shows the susceptibility calculated for 0≤β≤1 and α=1. Here, one can observe a transition from a pure Drude model β=1 to a resonant model with resonance frequency of $\omega =\omega_{p}$ (β=0). Again, the calculations become inaccurate in the regions of high absorption at ω→0 and near the resonance. Overall, one can conclude that the model allows for a very significant alteration of the dispersion relation while retaining good stability and accuracy.

**Figure 2:**
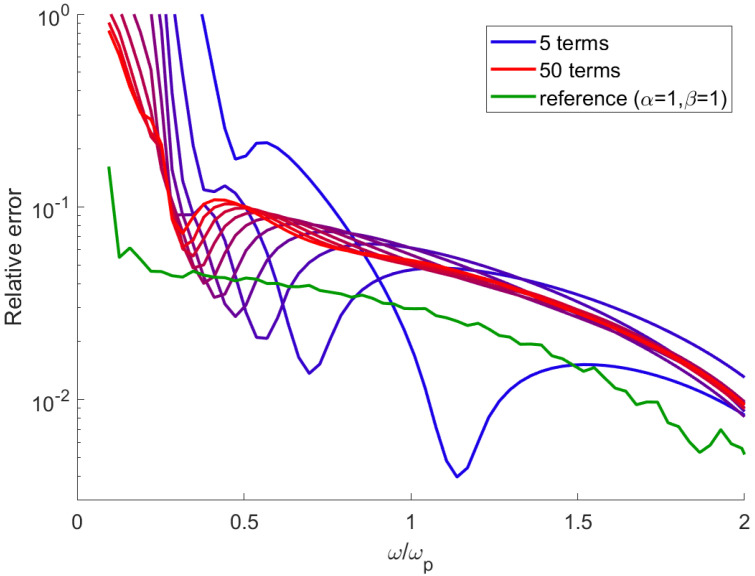
Relative error of the model for α=0.8, β=0.8. Standard Drude model (green line) added for reference.

An important factor for efficient implementation of the proposed model is the number of terms *N* in the sums in Equation (16). Figure 2 shows the relative error of the numerical susceptibility as a function of frequency, calculated with various numbers of the memory terms $P_{t-k}$. One can observe that in general the error initially quickly decreases with frequency for $\omega <0.5\omega_{p}$ with a further, slower decrease at higher ω. The number of terms has a high impact on the low frequency accuracy. In the solutions with low numbers of terms, the error has a significant minimum; the calculated values below and above the frequency where the minimum occurs are overestimated or underestimated, accordingly. This can be attributed to the so-called numerical dispersion [17], which introduces a small frequency-dependent term to the susceptibility regardless of the medium model. The effect is easily visible in Figure 1a for the case of α=1.5; the numerical results (dots) overestimate the susceptibility for $\omega <\omega_{p}$ and underestimate it beyond this frequency. In the majority of the spectral range, the error is greater than in the case of the standard Drude model (Figure 2, green line) by a factor of 2–3. Apart from the large minimum, the error also exhibits a slight oscillatory behavior, with the amplitude of oscillations reducing with an increasing number of terms. One may conclude that the minimum number of summation terms that provide satisfactory accuracy is N∼15, with relatively little gain from increasing *N* further. Moreover, the smooth error function with no short-term changes indicates that the model can be easily tuned to maintain a very high accuracy within a chosen, limited spectral range; in the standard FDTD implementation, it is straightforward to add a frequency-independent term $\epsilon_{\infty }$ to the dielectric susceptibility ϵ(ω)=1+χ(ω) [17], regardless of the medium model introduced with ADE. By doing this, one can shift the spectral region where the numerical results exactly match the theory. Alternatively, an additional Drude-like term can be introduced to ADE to counteract the numerical dispersion. Finally, due to the fact that parameters α and β can be changed continuously, one can optimize the accuracy by using slightly different values for theoretical and numerical calculations.

The stability and accuracy estimations for various values of parameters α, β and Δt are shown in Table 1.

In general, the approach is the most accurate for parameters α, β that are close to unity. For stable operation, the parameter 0<α<2, while 0≤β<1.3. The error is roughly proportional to the size of the time step, which needs to be chosen to obtain a desired compromise between the calculation accuracy and speed. As in the nondispersive, one-dimensional FDTD, the approach loses stability for Δt>1. One can see that the loss of stability is preceded by a significant increase in the error. Interestingly, for integer values α=1, β=2 the approach is stable despite the fact that β is significantly larger than its maximum stable value of ∼1.2.

The importance of the memory terms and hereditary properties of the medium decreases as the parameters α, β approach the limit of the standard Drude model, e.g. α=β=1; in such a case, the necessary number of memory terms for the accurate computation is reduced. In many applications, only a small modification of the medium is considered [16]. In this study, we focus on the frequency response of the modeled medium to monochromatic waves; the above-mentioned hereditary properties are more pronounced in the time domain studies, especially in the context of mechanical stress–strain relations and viscoelasticity [5,11,13,28]. In the field of electromagnetism, Gomez [14] has studied the step response of the fractional-derivative Drude model in the time domain, discussing the memory effects.

In the standard, two-dimensional implementation of FDTD, one has to define three scalar field values at every grid point (for example, two components of the magnetic field vector $H_{x},H_{y}$ and one component of the electric field $E_{z}$ perpendicular to the simulation plane) [17]. The inclusion of Drude or Drude–Lorentz dispersion models, as described in [27], adds the medium polarization *P*. Particularly, one current ($P_{t}$) and one previous ($P_{t-1}$) value of polarization is needed, resulting in 5 scalar values per grid point. The example implementation of the fractional Drude model with 12-term memory adds another 10 scalar values, increasing the memory requirement by a factor of 3. However, it should be noted that the increase is needed only for the part of the computational domain that contains the fractional model medium.

Finally, it should be mentioned that the coefficients $\alpha_{k}$, $\beta_{k}$ in the sums in Equation (16) need to be computed once for any given values of α, β and the calculation of the weighted sum in Equation (13) is essentially a discrete convolution operation, which can be subject to various numerical optimizations. An extensive discussion of the numerical application of convolution to calculate a fractional derivative is presented in [29].

Another advantage of the presented model is its tunability. By allowing adiabatic changes of α and β in the time domain simulation, one can achieve a dynamical tuning of the optical properties of the medium.

### 4.2. Wave Propagation in Fractional Medium

Dispersive properties of the medium described by the function χ(ω) influence the propagation of electromagnetic waves through the material. It directly affects the permittivity ϵ=1+χ, refraction index $n=\sqrt{\epsilon }$ and the value of the wave vector k=ωn/c. Assuming a harmonic wave with frequency ω and wavevector $\vec{k}$, from the relation (5) one obtains

(17) $$k^{2}=\frac{\omega^{2}}{c^{2}}\left(1+\frac{\omega_{p}^{2}}{\gamma_{\alpha }{\left(-i\omega \right)}^{\alpha +1}+\gamma_{\beta }{\left(-i\omega \right)}^{\beta }}\right).$$

The above relation is nontrivial for real parameters α, β due to the fact that both terms in the denominator introduce separate, frequency-dependent contributions to both real and imaginary parts of $\vec{k}$. One can simplify the problem by assuming that the medium is a small modification of the Drude model, with α=1 and β∼1; in such a case, in the high frequency limit one obtains

(18) $$k\approx \frac{\omega }{c}\left(1-\frac{\omega_{p}^{2}\gamma_{\alpha }\omega^{2}}{2\gamma_{\alpha }^{2}\omega^{4}}+i\frac{\omega_{p}^{2}\gamma_{\beta }\omega^{\beta }}{2\gamma_{\alpha }^{2}\omega^{4}}\right).$$

Thus, the imaginary part of *k* fulfills

(19) $$Im\;k∼\omega^{\beta -3}.$$

**Figure 3:**
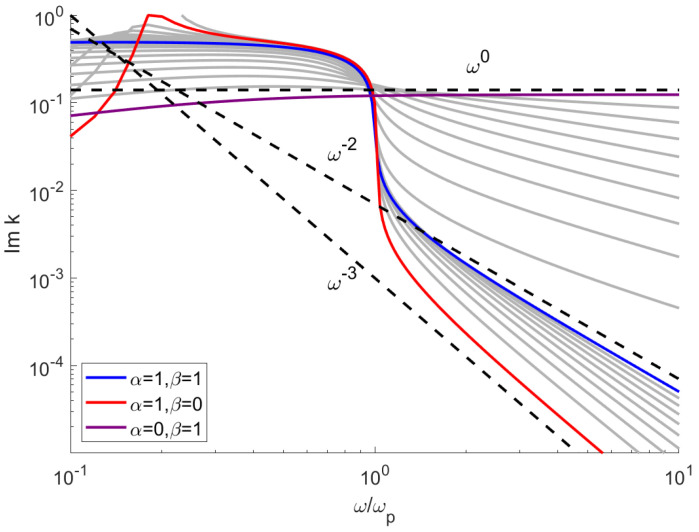
Imaginary value of the wave vector as a function of frequency, calculated for various values of α and β.

In a similar manner, one can derive the limit for α=0, which is $Im\;k∼\omega^{0}$. As mentioned in [7,9], there is a demand on dispersion models where the attenuation (which is proportional to Imk) follows a frequency power law. Figure 3 shows the numerically calculated imaginary part of the wave vector in the whole spectral range. The discussed limiting cases are shown with colored lines, while the transitional spectra with fractional values of α, β are shown in gray.

There are two distinct regions $\omega <\omega_{p}$ and $\omega >\omega_{p}$; below the plasma frequency, the susceptibility is negative and correspondingly ϵ≈0, $Re\;\vec{k}\approx 0$; the wavevector is thus purely imaginary, which corresponds to highly absorbed, evanescent waves. The parameter β has a negligible impact on the absorption in this range. Above the $\omega_{p}$, the absorption follows a power law (straight line), with the results consistent with Equation (19). The dependence on the parameter α is more complicated; the steep reduction of absorption at $\omega \approx \omega_{p}$ for α=1 becomes more gradual for smaller values of α. In the limit of α→0, the absorption becomes almost constant. In contrast to β, the parameter α has a significant impact on the imaginary part of *k* in the region $\omega <\omega_{p}$. While waves with such frequencies are highly attenuated when propagating through the medium, the negative value of permittivity (see Figure 1) allows for formation of surface plasmons. These collective electron oscillations are highly sensitive to changes in medium properties [30], which makes them a particularly promising field of study, where the proposed model could be applied. One of the prospective applications of the fractional derivative model and resulting power-law dissipation are metallic nanoparticle chains [9].

The above mentioned power-law dependence extends to other material functions such as conductivity

(20) $$\sigma =\epsilon_{0}\omega Im\;\chi .$$

**Figure 4:**
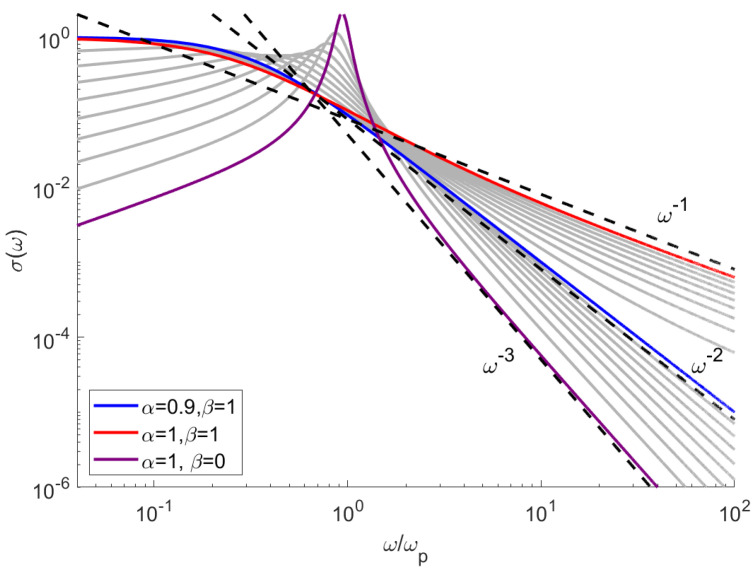
The electric conductivity as a function of frequency, calculated for various values of α and β.

In the paper [16] the author proposed a fractional-derivative model based on Equation (2), which is transformed to a dimensionless, first-order differential equation describing particle velocity. Our model is consistent with the results in [16] when one sets β=1 and α≈1, resulting in a small modification of the standard Drude model. Calculation results for such parameters are shown in Figure 4. One can see that while a small modification of α has a little impact on the σ for $\omega ∼\omega_{p}$, it dramatically changes the high-frequency behavior of the medium. As the order of the fractional derivative decreases, the slope of the high-frequency asymptote increases, which agrees with [16]. The impact of β is much smaller in the high-frequency range, but is more significant for $\omega <\omega_{p}$.

### 4.3. Application to Soft Tissue

**Figure 5:**
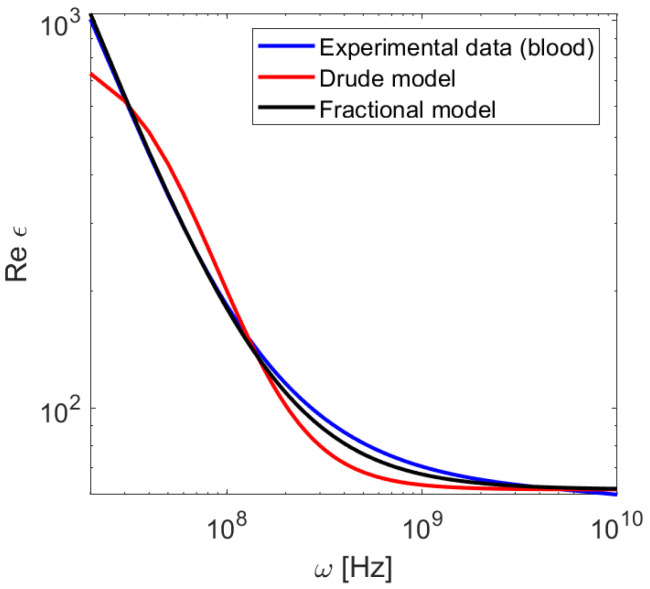
Dielectric susceptibility of blood as a function of frequency.

As a practical test of the usefulness of the proposed model, we take the data from [26], where the susceptibility and conductivity of 17 soft tissue types has been presented. Specifically, we focus on the dispersive characteristics of blood in the region of $10^{7}$–$10^{10}$ Hz. A characteristic feature of this medium is anomalous dispersion in a wide range of frequencies, e.g., ∂ϵ/∂ω<0, which is a result of many relaxation processes taking place in this frequency range [21]. The standard Drude model (3) can be used to describe such a medium by taking $\omega_{p}^{2}<0$. However, its accuracy is limited to a small part of the spectrum (Figure 5). The greater flexibility of the Equation (4) allows for a much better fit. The calculated fitting parameters are $\epsilon_{\infty }=61.6$, σ=1.33, $\omega_{p}=10^{7}$ Hz, $\gamma_{a}=13$, $\gamma_{b}=0.99$, α=0.31, β=0.04. We have confirmed that FDTD results are stable and consistent with the model despite the negative value of $\omega_{p}^{2}$. It should be noted that the Cole–Cole model used in [26] to fit the experimental data consisted of two separate terms of the form (8). This, in turn, necessitates the introduction of two auxiliary arrays and several additional calculation steps in FDTD computation [19]. As shown in Figure 2, our model provides accuracy in the order of 1% with the introduction of 3 additional arrays (5 terms in total).

## 5. Conclusions

A novel, two-parameter modification of the Drude model based on the Grunwald–Letnikov fractional derivative has been presented. The analytical formulas for basic optical functions such as susceptibility have been derived and discussed in the context of wave propagation in the medium. A numerical implementation of the model in the FDTD approach has been realized, expanding a well-known ADE method and taking advantage of efficient discrete convolution computation. The numerical complexity and accuracy of the approach was discussed and the limits of its stability have been established. The results indicate that the proposed model is highly flexible and applicable to a wide variety of optical and plasmonic systems, allowing for modeling of other modified Drude models, as well as many fractional-derivative-based descriptions of complex dielectrics, including soft tissues. This property makes it particularly promising for application in electromagnetic biosensors.

## Figures and Tables

**Table 1:** Mean relative error η, in percent, between theoretical and simulated susceptibility, calculated in the range $\omega \in (0.3\omega_{p},1\omega_{p})$, for various values of parameters α, β and the time step Δt; infinity (∞) indicates that the approach loses stability and no results are obtained.

α∈〈0,2〉, β=1, Δt=0.5																					
α	0	0.1	0.2	0.3	0.4	0.5	0.6	0.7	0.8	0.9	1	1.1	1.2	1.3	1.4	1.5	1.6	1.7	1.8	1.9	2
η	∞	89	37	26	19	16	13	11	9	6	4	3	1	2	5	9	16	27	48	110	∞
β∈〈0,1〉, α=1, Δt=0.5																					
β	0	0.1	0.2	0.3	0.4	0.5	0.6	0.7	0.8	0.9	1	1.1	1.2	1.3	1.4	1.5	1.6	1.7	1.8	1.9	2
η	9	11	12	13	13	12	12	12	11	10	9	12	690	∞	∞	∞	∞	∞	∞	∞	117
Δt∈〈0.05,1.1〉, α=1, β=1																					
Δt	0.05	0.1	0.15	0.2	0.25	0.3	0.35	0.4	0.45	0.5	0.55	0.6	0.65	0.7	0.75	0.8	0.85	0.9	0.95	1	1.1
η	0.5	1	1	2	2	3	3	3	4	4	6	6	7	8	9	10	11	12	14	16	∞
Δt∈〈0.05,1.1〉, α=1.2, β=0.9																					
Δt	0.05	0.1	0.15	0.2	0.25	0.3	0.35	0.4	0.45	0.5	0.55	0.6	0.65	0.7	0.75	0.8	0.85	0.9	0.95	1	1.1
η	0.5	0.8	0.7	0.7	0.6	0.5	0.1	0.4	1	2	3	4	5	6	7	9	10	12	14	17	∞

## Data Availability

Not applicable.
